# Supplementary material for: A simian-adenovirus-vectored rabies vaccine suitable for thermostabilisation and clinical development for low-cost single-dose pre-exposure prophylaxis
Source: PLoS Negl Trop Dis. 2018 Oct 29;12(10):e0006870. doi: 10.1371/journal.pntd.0006870 (PMC6224154; doi:10.1371/journal.pntd.0006870)
Supplement: S1 Fig — Panel A shows ELISA-measured antibody induction following immunization by AdHu5, ChAd63, and ChAdOx1 adenovirus-vectored rabies vaccines as compared to Rabipur and Nobivac Rabies IRVs. Individual mice are represented by points, with lines linking the week 4 (left, circles) and week 12 (right, crosses) measurements for each animal. P-values obtained from repeat measures two-way ANOVA were as tabulated in Panel B. For all vaccines, P<0.0001 for effect of matching of repeated measures. (PDF) [file pntd.0006870.s001.pdf]

Supplementary Figure 1

A

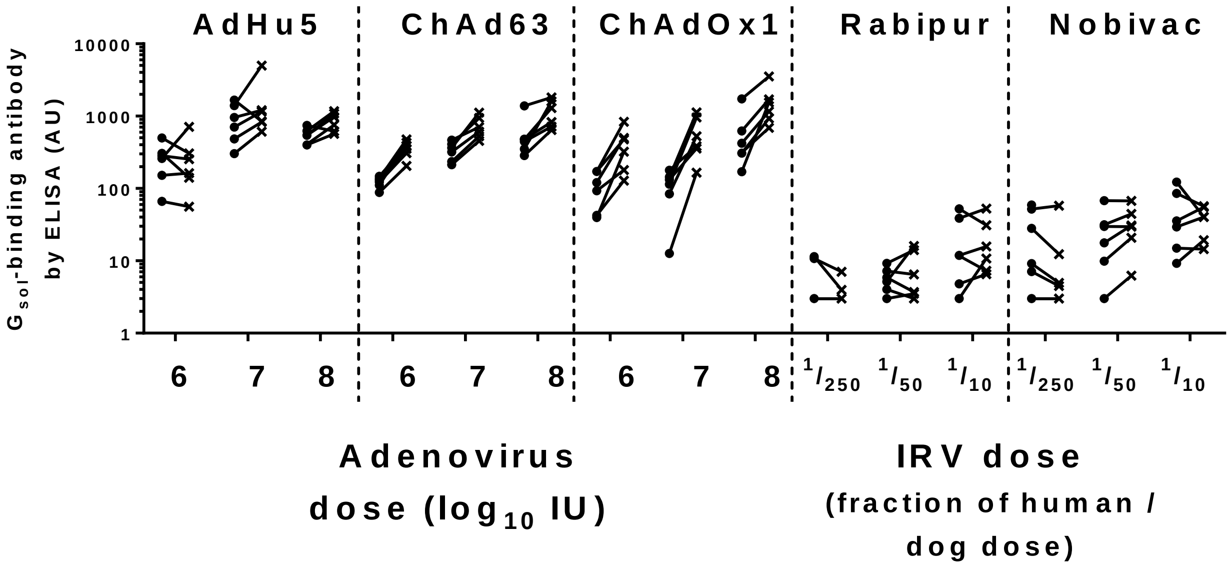

B

	Parameter		
Vaccine	***Time***	***Dose***	***Interaction***
AdHu5 RabG	*0.057*	*0.0008*	*0.204*
ChAd63 RabG	*<0.0001*	*<0.0001*	*0.212*
ChAdOx1 RabG	*<0.0001*	*0.003*	*0.238*
Rabipur	*0.819*	*0.019*	*0.358*
Nobivac Rabies	*0.947*	*0.148*	*0.074*
